# Morphometric study of the atlantooccipital and the lateral atlantoaxial joints in small breed dogs with and without atlantoaxial instability

**DOI:** 10.3389/fvets.2025.1699281

**Published:** 2026-02-09

**Authors:** Felix Pack, Valerie Hungerbühler, Bastien Planchamp, Franck Forterre, Christina Precht

**Affiliations:** 1 Division of Small Animal Surgery, Department of Clinical Veterinary Science, Vetsuisse Faculty, University of Bern, Bern, Switzerland; 2 Veterinary Public Health Insitute, Department of Clinical Research and Veterinary Public Health, Vetsuisse Faculty, University of Bern, Bern, Switzerland; 3 Division of Clinical Radiology, Department of Clinical Veterinary Science, Vetsuisse Faculty, University of Bern, Bern, Switzerland

**Keywords:** atlantooccipital dysplasia, computed tomography, craniovertebral junction, morphometry, small breed dog, toy breed dog

## Abstract

**Introduction:**

The combination of atlantoaxial instability (AAI) with several abnormalities and malformations of the craniovertebral junction in small breed dogs has been described, however there is limited knowledge regarding morphometric alterations of the synovial joints in this region. This study aimed to evaluate the morphometric differences of the lateral atlantoaxial and the atlantooccipital joints between dogs with and without AAI using computed tomography (CT) scans.

**Materials and methods:**

This retrospective observational case–control study analyzed multiplanar reconstructed CT scans of 33 small and toy breed dogs (11 with AAI and 22 controls without AAI). The depth-to-length ratios of the articular surfaces of the atlantooccipital and lateral atlantoaxial joints were measured in dorsal and sagittal reconstructions and tested for significant differences between AAI and control groups.

**Results:**

The depth-to-length ratio of the occipital condyles in sagittal (*p* < 0.001) and dorsal planes (*p* < 0.007), as well as the facies articularis cranialis of the atlas in sagittal plane (*p* < 0.001), were significantly smaller in the AAI group, indicating that the occipital condyle and the cranial articular surface of the atlas were more shallow in the AAI group compared to the control group. The depth-to-length ratios of the lateral atlantoaxial joint surfaces were generally lower than those of the atlantooccipital joint, consistent with a flatter joint conformation, and some differences between AAI and control dogs were detected; however, these patterns were not consistent across planes.

**Discussion and conclusion:**

The atlantooccipital joint surfaces were more shallow in small breed dogs with AAI compared to controls. These results suggest that a certain degree of atlantooccipital joint dysplasia may be more commonly present in dogs with AAI, whereas morphometric alterations of the lateral atlantoaxial joints appear more complex and less consistently differentiated between groups.

## 1 Introduction

Atlantoaxial instability (AAI) is a condition predominantly affecting small dog breeds, particularly toy breeds, and is accompanied by various abnormalities of the craniovertebral junction (CVJ). In many cases, traumatic factors are reported in combination with congenital anomalies (1–3).

In addition to clinical evaluation and neurological assessment, advanced imaging techniques, particularly Magnetic Resonance Imaging (MRI) and Computed Tomography (CT), are integral for diagnosing AAI. These imaging modalities allow for the identification of dorsal subluxation of the Dens axis, which can lead to spinal cord compression and subsequent neurological deficits (4–8).

Ligaments are key stabilizing structures of the craniovertebral junction, and their malformation or laxity can contribute to atlantoaxial instability (9–12). Particularly, the absence of the transverse ligament, which runs between the lateral masses of the atlas and holds the dens of the axis in position, has been observed in AAI (13).

Furthermore, the muscular components, particularly the paraspinal muscles, contribute significantly to the stability of the craniovertebral junction. A previous study demonstrated the potential role of these muscles in maintaining spinal stability in dogs with spinal instability (14). Moreover, Lee et al. (15) suggested that chronic changes in the musculature may also play an additional role in maintaining stability in the CVJ region.

While the role of ligaments and muscles in the stability of the CVJ has been investigated, the morphological characteristics of the CVJ, particularly the atlantooccipital and lateral atlantoaxial joints, have received relatively little attention. Despite the extensive research on pathologies of the median atlantoaxial joint, morphometric alterations in the synovial joints, especially the lateral atlantoaxial joints, remain largely unexplored. Moreover, potential dysplasia or malformation in these joints in dogs with AAI has not been systematically investigated.

The atlantooccipital joint and the atlantoaxial joint, which is primarily involved in the pathophysiology of AAI, are critical to the stability of the craniovertebral junction. Both joint spaces are communicating and share a synovial cavity. The atlantooccipital joint, formed by the Condyli occipitales and the Foveae articulares craniales of the atlas, is responsible for the flexion and extension of the head. The atlantoaxial joint is a more complex structure, consisting of one median and two lateral joint parts. The median part of the atlantoaxial joint is formed by the Dens axis and the Fovea dentis of the atlas, while the lateral parts of the atlantoaxial joint are formed by the Foveae articulares caudales of the atlas and the Facies articulares ventrales of the axis, allowing for rotational movement of the head. While abnormalities of the median atlantoaxial joint, such as dens anomalies (e.g., hypoplasia or aplasia), have been well-documented as contributing to AAI (3, 9, 16–18) other abnormalities affecting the craniovertebral junction, such as incomplete ossification of the atlas, occipital dysplasia, atlantooccipital overlapping, and Chiari-like malformations, are also well recognized as destabilizing factors in small breed dogs (19–25).

The hip joint, due to its anatomical structure and the critical role of conformation in joint stability, serves as a well-studied example of how conformational abnormalities can predispose joints to instability. Hip dysplasia, a developmental defect, leads to progressive degeneration of the joint, ultimately resulting in pain and loss of function. This example underscores the importance of joint morphology in maintaining stability and function (26). However, the potential role of abnormal morphology of the Condyli occipitales in CVJ instability has not yet been documented in veterinary medicine. In human medicine, studies have shown that abnormalities in the Condyli occipitales can be linked to disruptions in the normal embryological development of the CVJ (27). Furthermore, occipital condyle aplasia has been observed in human patients with Chiari malformation (28). A veterinary study by Fernandes et al. (29) found that cervical vertebral malformations in dogs are rare and often remain undiagnosed.

In human medicine, one case report described deformations in the atlantooccipital joint possibly secondary to or related to AAI (30). Additionally, a study examining patients with Chiari malformation type 1 found that the atlantooccipital joints were significantly flatter compared to those in healthy controls (31). A further study by Ma et al. (32) conducted a morphometric analysis of the lateral atlantoaxial joints in human patients with old type II odontoid fractures and atlantoaxial dislocation. The findings suggested that morphological changes in these joints could become more pronounced as the disease evolves (32).

In the present study, we aimed to conduct a morphometric analysis using multiplanar reconstructions from CT imaging to evaluate the morphological characteristics of the atlantooccipital and lateral atlantoaxial joints in small breed dogs with and without AAI. We hypothesize that there are significant differences in the morphometry of the atlantooccipital and/or lateral atlantoaxial joints between dogs with and without AAI, suggesting that these structures may be associated with the development or severity of the instability.

## 2 Materials and methods

### 2.1 Sample inclusion criteria

#### 2.1.1 AAI Group

A retrospective review of medical records was conducted for toy and small breed dogs with CT scans of the craniocervical region performed between 2009 and 2020. Data extracted from medical records included breed, gender, age, body weight, clinical signs, and CT images of the craniocervical region. Dogs were included in the AAI group if they had a clinical diagnosis of AAI, confirmed through neurological and clinical examination, and exhibited subjective imaging features indicative of AAI on CT scans of the CVJ at the time of presentation. Only small or toy breed dogs (<5 kg body weight) were included. Dogs not meeting these criteria were excluded.

#### 2.1.2 Control group

Control dogs were small and toy breed dogs euthanized for reasons unrelated to the study and collected over several years. The cadavers were stored at −25 °C and thawed at room temperature for 24 h prior to imaging. Only skeletally mature dogs without clinical signs of AAI or structural anomalies in the atlantoaxial joint (as assessed by CT) were included to represent normal joint morphology. Dogs with any atlantoaxial anomalies were excluded to ensure a morphologically normal comparison group. All control dogs were small or toy breeds (<5 kg body weight), and breed, gender, and body weight were recorded. The exact age and reason for euthanasia were unknown.

### 2.2 Diagnostic imaging

CT scans of the head–neck region were performed using a 16-slice CT scanner (Philips Brilliance, Philips AG Healthcare, Zürich, Switzerland). High-resolution scans were acquired with the following settings: 120 kVp, 180 mAs, 0.8 mm slice thickness, 0.4 mm increment, and a spatial resolution of 0.4 mm in the *z*-axis and 0.1 mm in the *x*- and *y*-axes. The bone window was set to a window level of 800 Hounsfield units (HU) and a window width of 2000 HU. Morphometric measurements were performed using the Deep Unity Diagnost software (Dedalus Healthcare Systems Group, Bonn, Germany).

### 2.3 Measurements

**Figure 1:**
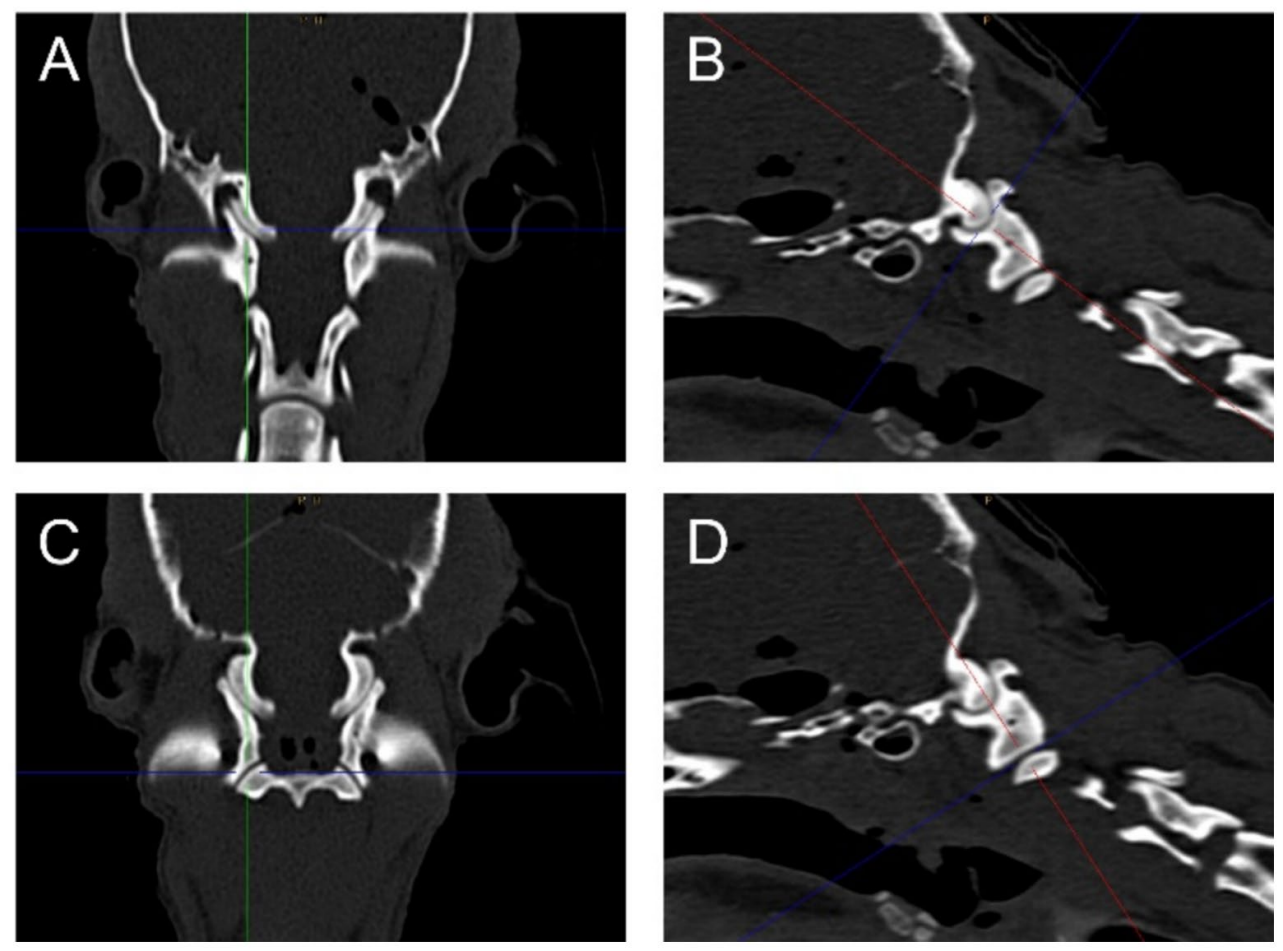
Dorsal **(A,C)** and parasagittal **(B,D)** reconstructions of the right side of the atlantooccipital **(A,B)** and the lateral atlantoaxial **(C,D)** joints of an example dog from the control group illustrating the reconstructed planes, in which the measurements were performed.

Measurements were obtained from multiplanar reconstructions, generating transverse, dorsal, and sagittal planes through the atlantooccipital and lateral atlantoaxial joints. To ensure consistent reconstructions of the joint surfaces, the crosshair of the multiplanar reconstruction tool was first centered at the level of the transverse processes of the atlas for the dorsal and transverse planes, and centrally through the two parts of the atlas for the sagittal plane. The crosshair was then moved parasagittally to the center of the atlantooccipital and lateral atlantoaxial joints, respectively, and adjusted to align parallel to the joint surface in a modified, slightly oblique sagittal or dorsal plane to optimally visualize the length and depth of the respective joint surfaces (Figure 1). The length and depth of the joint surfaces were measured, and the depth-to-length ratio was calculated as a morphometric parameter to assess joint curvature (31).

**Figure 2:**
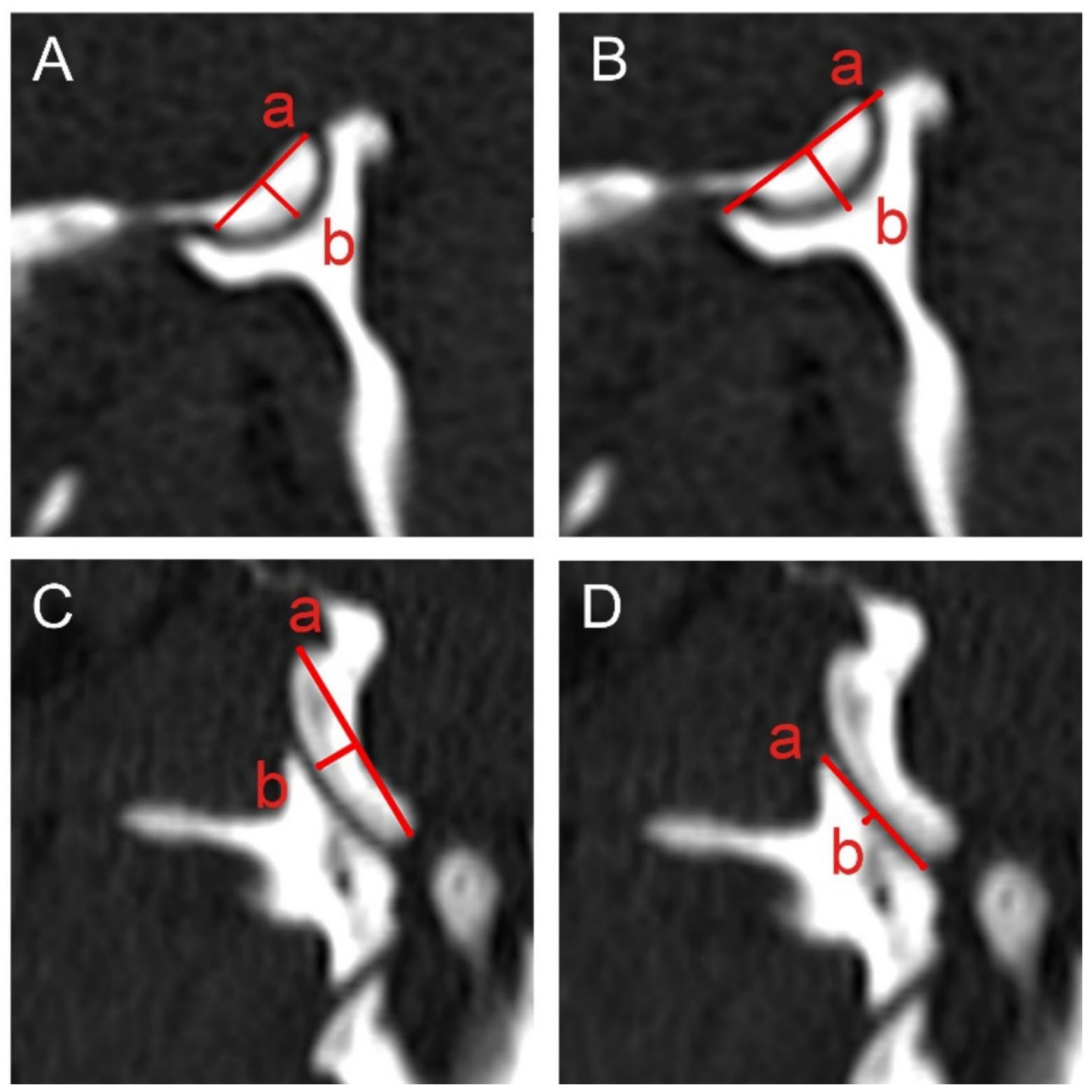
Measurements of the atlantooccipital joint in the sagittally **(A,B)** and dorsally **(C,D)** reconstructed planes. Measurements of the length (a) and depth (b) of the condylus occipitalis **(A,C)** and fovea articularis cranialis atlantis **(B,D)** are illustrated.

**Figure 3:**
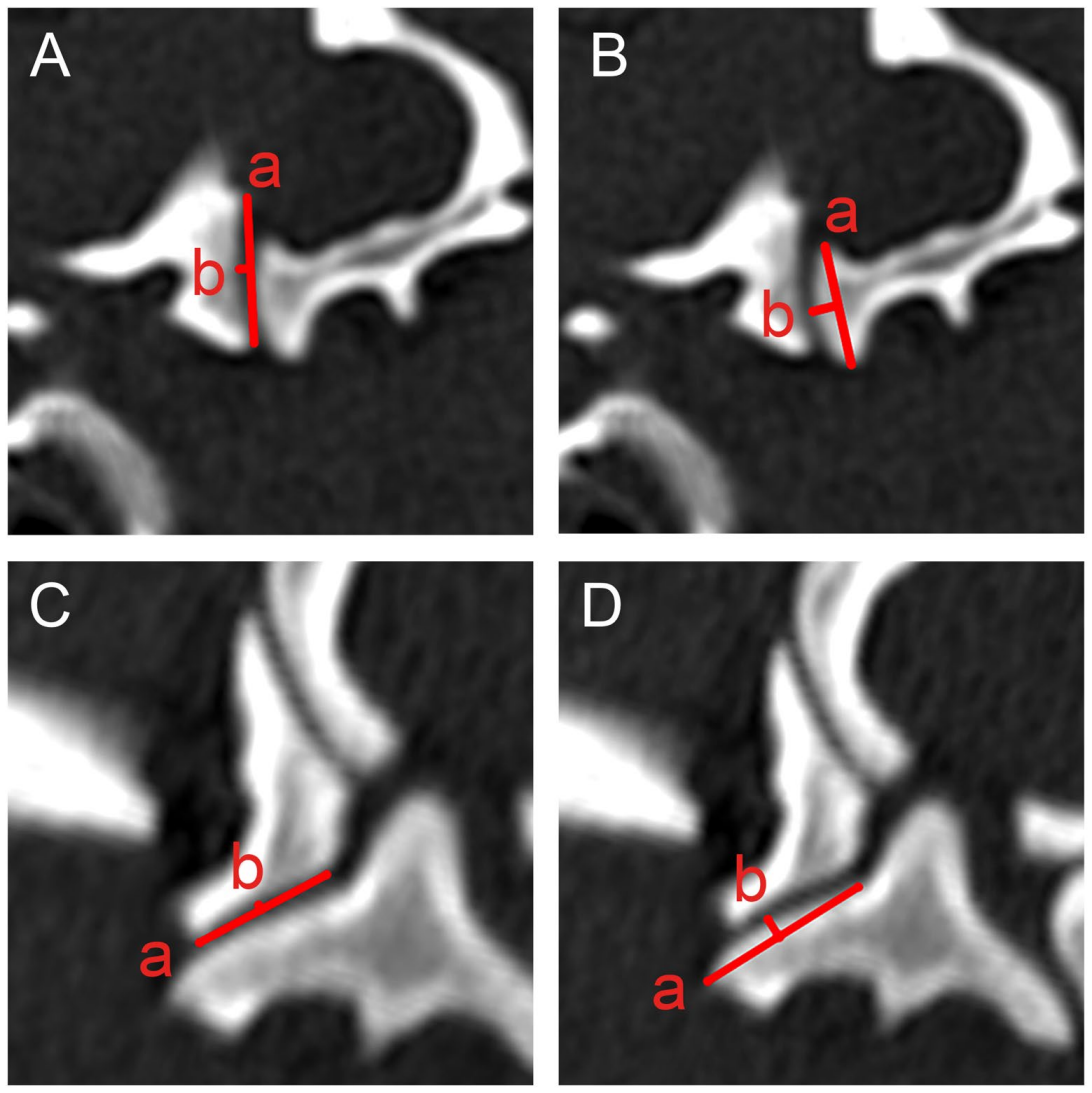
Measurements of the lateral atlantoaxial joints in the sagittally **(A,B)** and dorsally **(C,D)** reconstructed planes. Measurements of the length (a) and depth (b) of the fovea articularis caudalis atlantis **(A,C)** and the facies articularis ventralis axis **(B,D)** are illustrated.

The measurement protocols for the atlantooccipital (Figure 2) and lateral atlantoaxial (Figure 3) joints are similar for all four joint surfaces (Condylus occipitalis, Fovea articularis cranialis atlantis, Fovea articularis caudalis atlantis, and Facies articularis ventralis axis). For both joints, the length and depth were measured in both adjusted sagittal and dorsal planes using multiplanar reconstructions.

- Length: For all joint surfaces, the length was measured as the distance between the most craniodorsal and caudoventral edges of the respective articular surface in the sagittal plane, and the axial and abaxial edges in the dorsal plane, respectively.
- Depth: Depth was measured perpendicular to the length, from the midpoint of the length line to the highest or lowest point of the joint surface, depending on the surface being measured.

Measurements were performed by a small animal surgery specialty intern under the supervision of a board-certified veterinary radiologist. Observers were blinded to group information.

### 2.4 Statistical analysis

All statistical analyses were conducted using SPSS Statistical Software (IBM SPSS Statistics, Version 29.0.2.0, IBM Switzerland Ltd., Zürich, Switzerland). For all hypothesis tests, *p*-values below 0.05 were considered statistically significant. Descriptive statistics were computed for all data, and the normality of the continuous variables was first assessed visually using histograms and then tested with the Kolmogorov–Smirnov test. The depth and length measurements, as well as the depth-to-length ratios of the left and right sides, were compared and tested for side differences separately for the control and AAI groups using the Wilcoxon signed ranks test for related samples. To assess differences between the control and AAI groups for signalment variables (such as gender and weight) and morphometric variables (depth and length measurements and depth-to-length ratios), the Chi-square test was employed for categorical and the Wilcoxon-Mann–Whitney test for metric variables. Effect sizes (r) were calculated and interpreted as small (*r* ~ 0.10), medium (*r* ~ 0.30) or large (*r* ~ 0.50). Based on the observed difference in the depth-to-length ratios between AAI and control dogs, we performed a post-hoc sample size calculation using an independent-samples *t*-test (two-sided, *α* = 0.05, power = 0.80).

## 3 Results

A total of 33 patients were included in this study. Of these, 11 dogs had AAI (AAI group), and 22 dogs were not affected by AAI (control group).

Of the 11 dogs with AAI (AAI group), the most common breeds were Chihuahua (*n* = 3), Yorkshire Terrier (*n* = 3) and Pomeranian (*n* = 2). Additionally, one dog from each of the following breeds was included: Bolonka Zwetna, Italian Greyhound, Bichon frisé. The median body weight of the AAI group was 2.10 (1.20–4.40) kg, and the average age was 3 years. The group comprised 6 female and 5 male dogs.

The control group consisted of 22 dogs. The most frequently represented breeds were Chihuahua (*n* = 8) and Yorkshire Terrier (*n* = 7), followed by Maltese (*n* = 2), Pomeranian (*n* = 2), Pinscher (*n* = 2), and Japan Chin (*n* = 1). The median body weight of the control group was 1.35 (0.59–4.70) kg. The exact age of the dogs at the time of imaging is unknown, but all dogs were at least 9 months old, as indicated by the closure of the growth plates in the cervical spine. The control group included 8 female and 14 male dogs.

There was no significant difference between the AAI and the control group concerning the gender (*p* = 0.319). Although the median weight of the AAI group was larger than in the control group, the difference was not significant (*p* = 0.07).

Results of the depth-to-length ratios of the articular surfaces of the atlantooccipital and lateral atlantoaxial joints are summarized in Table 1. Several variables were found to be non-normally distributed, therefore non-parametric tests were applied for hypothesis testing. Except for the depth-to-length ratio of the Condylus occipitalis and the Facies articularis ventralis axis in the AAI group, no significant differences between the left and the right sided measurements in both the control and the AAI groups were observed, so the data from the left and right sides were pooled for further analysis of differences between the control and AAI groups (Supplementary Table 1).

**Table 1:** Results of the morphometric measurements and the *p*-value of the Wilcoxon-Mann–Whitney test evaluating for differences between the control (control) and AAI (AAI) groups.

Measurement	Median		*p* value	Mean		Standard deviation		Minimum		Maximum	
	Control	AAI		Control	AAI	Control	AAI	Control	AAI	Control	AAI
CO D/L Ratio Sag	0.43	0.31	0.001	0.41	0.32	0.09	0.08	0.19	0.19	0.55	0.53
CO D/L Ratio Dor	0.20	0.15	0.007	0.19	0.16	0.05	0.05	0.09	0.09	0.29	0.29
FACraAt D/L Ratio Sag	0.35	0.21	< 0.001	0.34	0.23	0.05	0.06	0.20	0.13	0.44	0.33
FACraAt D/L Ratio Dor	0.07	0.07	0.124	0.08	0.07	0.03	0.02	0.03	0.02	0.20	0.12
FACauAt D/L Ratio Sag	0.05	0.10	0.001	0.06	0.10	0.03	0.05	0.01	0.11	0.04	0.18
FACauAt D/L Ratio Dor	0.10	0.07	0.001	0.10	0.08	0.03	0.03	0.07	0.02	0.18	0.15
FAVenAx D/L Ratio Sag	0.18	0.19	0.229	0.18	0.22	0.03	0.09	0.13	0.15	0.27	0.45
FAVenAx D/L Ratio Dor	0.13	0.16	0.005	0.13	0.16	0.02	0.05	0.08	0.07	0.17	0.27

CO, Condylus occipitalis; FACraAt, Fovea articularis cranialis atlantis; FACauAt, Fovea articularis caudalis atlantis; FAVenAx, Facies articularis ventralis axis; D/L, depth-to-length; Sag, sagittal plane; Dor, dorsal plane.

**Figure 4:**
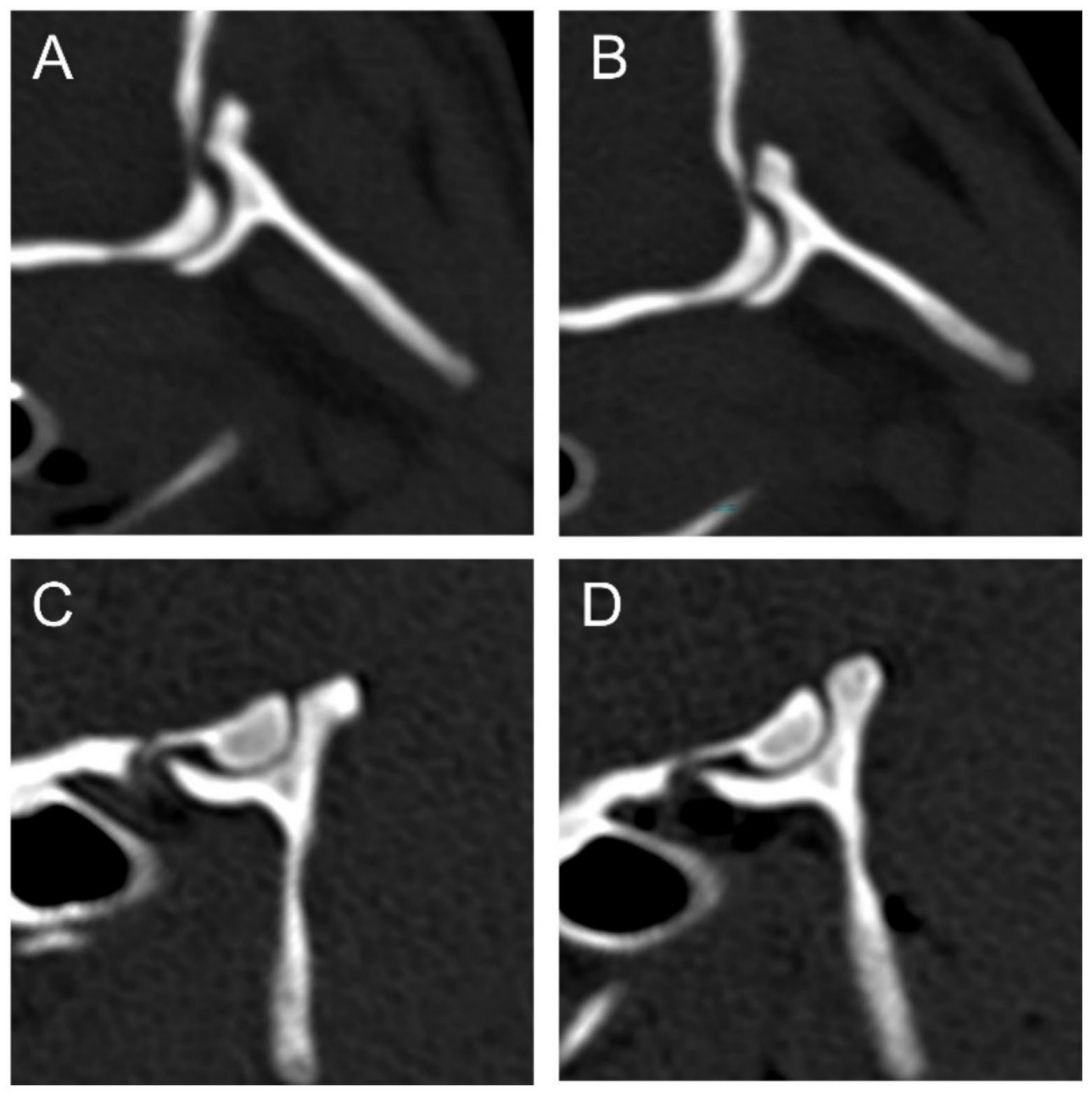
Sagittal reconstructions of the atlantooccipital joint of an example dog from the AAI **(A,B)** and the control **(C,D)** group illustrating that the joint surfaces are flatter in the dog from the AAI group **(A,B)**.

**Figure 5:**
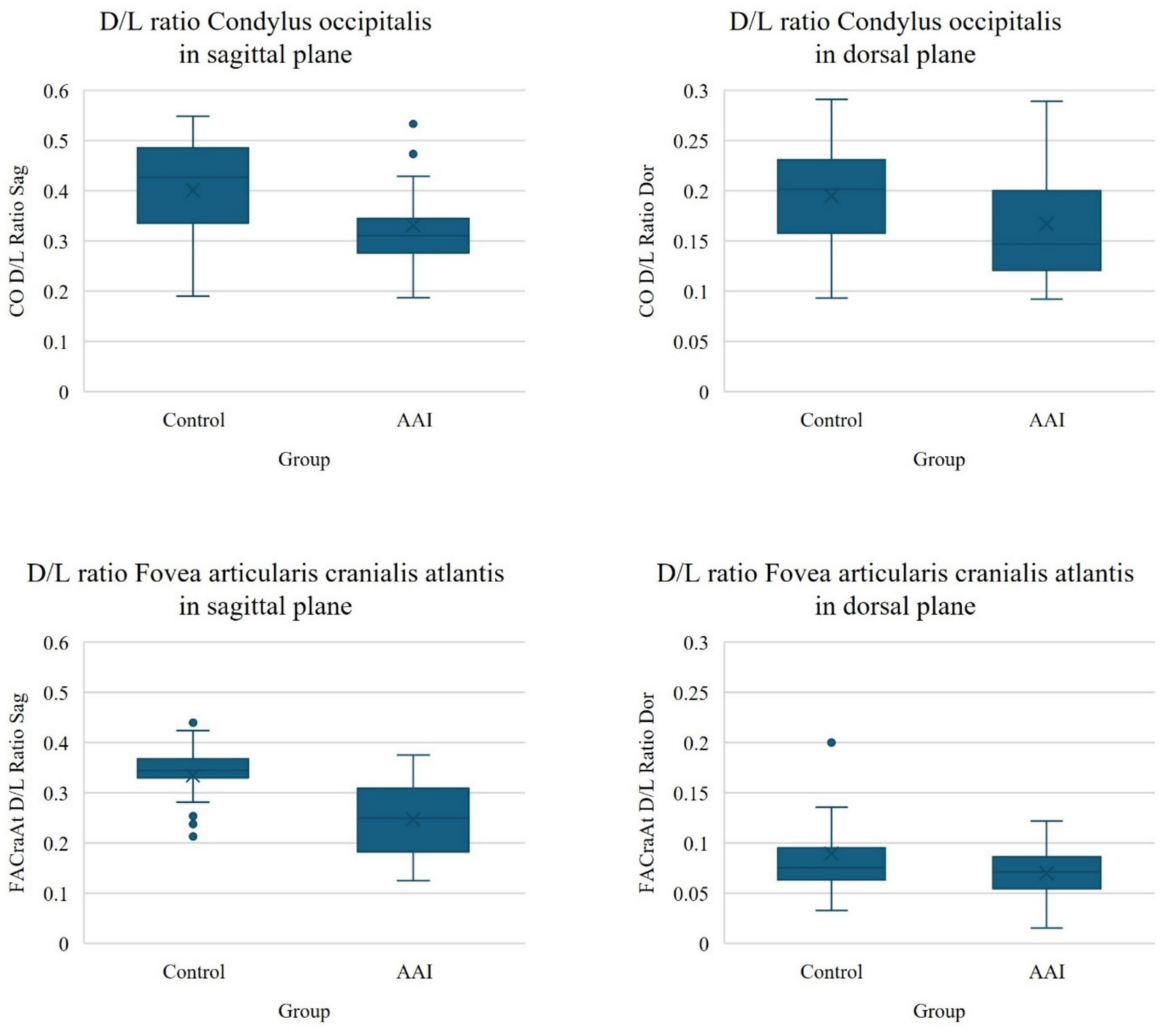
Box plots of the depth-to-length (D/L) ratios of the atlantooccipital joint in the control and AAI groups, including the Condylus occipitalis (CO) and the Fovea articularis cranialis atlantis (FACraAt) in sagittal (Sag) and dorsal (Dor) planes.

In the atlantooccipital joint, the depth-to-length ratios of both joint surfaces in both groups were larger in the sagittal compared to the dorsal plane, indicating that the primary curvature of the joint occurs in the sagittal plane. The depth-to-length ratio of the Condylus occipitalis was significantly smaller in the AAI group compared to the control group and had a medium-to-large effect size, with a median value of 0.31 and 0.43, respectively, in the sagittal plane (*p* = 0.001, *r* = 0.58) and a median value of 0.15 and 0.20, respectively in the dorsal plane (*p* = 0.007, *r* = 0.47). Also, the depth-to-length ratio of Fovea articularis cranialis atlantis was significantly smaller in the AAI group compared to the control group and showed a large effect size, with a median value of 0.21 and 0.35, respectively, in the sagittal plane (*p* < 0.001, *r* = 0.95). The post-hoc sample size calculation indicated that the required sample size to detect these differences was lower than the actual sample size in the sagittal plane and approximately equal to the actual sample size in the dorsal plane. In the dorsal plane, both groups had a median depth-to-length ratio of 0.07 for the Fovea articularis cranialis atlantis with no significant difference despite a small-to-medium effect size (*p* = 0.124, *r* = 0.28). The post-hoc sample size estimation indicated that a substantially larger sample would be required to detect a true difference of this magnitude, if such a difference indeed exists. Figure 4 illustrates the more shallow conformation of the atlantooccipital joint in the sagittal plane in example dogs from the AAI and control groups. Figure 5 shows the box plots of the depth-to-length ratios of the atlantooccipital joint. Supplementary Table 2 summarizes the post-hoc sample size calculation.

**Figure 6:**
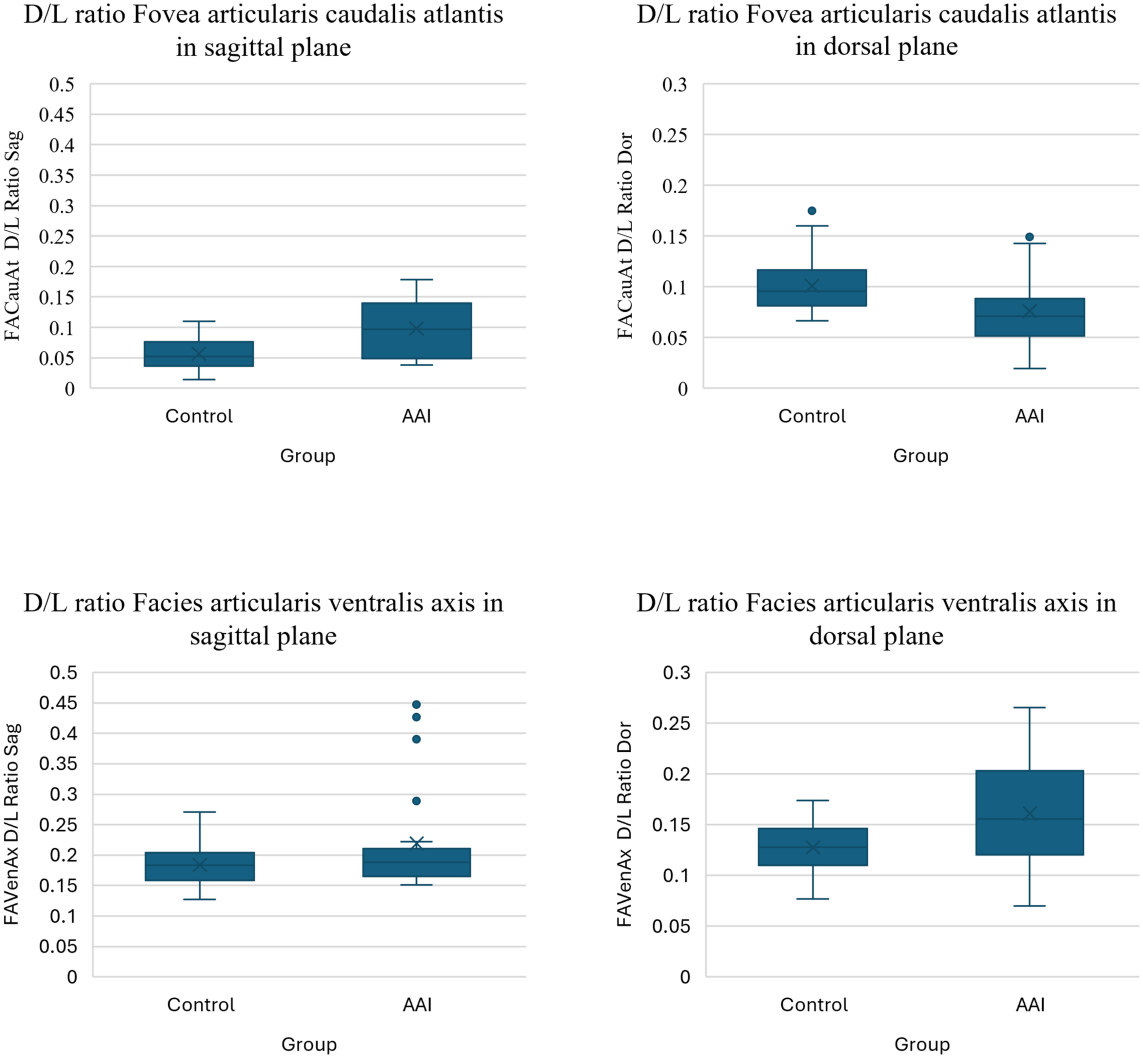
Box plots of the depth-to-length (D/L) ratios of the lateral atlantoaxial joint in the control and AAI groups, including the Fovea articularis caudalis atlantis (FACauAt) and the Facies articularis ventralis axis (FAVenAx) in sagittal (Sag) and dorsal (Dor) planes.

In the atlantoaxial joint, the depth-to-length ratios of both the Fovea articularis caudalis atlantis and Facies articularis ventralis axis were generally smaller than in the atlantooccipital joint in both the sagittal and dorsal planes. In the sagittal plane, the depth-to-length ratios of both the Fovea articularis caudalis atlantis and the Facies articularis ventralis axis were larger in the AAI group compared to the control group, however, this was significantly different and had a large effect size only for the atlas (*p* = 0.001, *r* = 0.58) with a median value of 0.10 and 0.05, respectively, but not for the axis (*p* = 0.229, *r* = 0.21), with a median value of 0.19 and 0.18, respectively. In the dorsal plane, the depth-to-length ratio of the Fovea articularis caudalis atlantis was significantly smaller in the AAI group compared to the control group, with a median value of 0.07 and 0.10, respectively, (*p* = 0.001, *r* = 0.59) and larger in the Facies articularis ventralis axis, with a median value of 0.16 and 0.13, respectively, (*p* = 0.005, *r* = 0.49). Both measurements showed a large effect size. The post-hoc sample size calculation indicated that the required sample size to detect these differences was approximately equal to the actual sample size for the Fovea articularis caudais atlantis, but a larger sample would be required to detect a true difference of this magnitude in the Facies articularis ventralis axis, if such a difference indeed exists. Figure 6 shows the box plots of the depth-to-length ratios of the atlantoaxial joint. Supplementary Table 2 summarizes the post-hoc sample size calculation.

## 4 Discussion

This study aimed to investigate the presence of morphological changes in the joint surfaces of the craniovertebral junction (CVJ) in small breed dogs with and without atlantoaxial instability (AAI) by comparing the depth-to-length ratios of the atlantooccipital and lateral atlantoaxial joints. A simple method to assess the curvature of these joints using reconstructed CT images was applied. Our results support the hypothesis that there are significant differences in the morphometry of the atlantooccipital joints between dogs with and without AAI, suggesting that these structural alterations may be associated with the development or severity of the instability.

In the atlantooccipital joint, we observed that the depth-to-length ratio was significantly lower in the AAI group, indicating that the occipital condyles (Condylus occipitalis) and the cranial articular surfaces of the atlas (Fovea articularis cranialis atlantis) were shallower in affected dogs. This difference was most pronounced in the sagittal plane, which is likely attributable to the joint’s anatomical configuration: the primary curvature of the atlantooccipital joint occurs sagittally, whereas the dorsal plane exhibits comparatively less curvature, supported by the generally higher depth-to-length ratios observed in the sagittal plane compared to the dorsal plane. These findings are consistent with the physiological biomechanics of the atlantooccipital joint, where flexion-extension is the predominant motion, facilitated by a sagittally cup-shaped morphology—characterized by a convex Condylus occipitalis articulating with a concave Fovea articularis cranialis atlantis (33).

In human medicine, a flatter atlantooccipital joint conformation has been associated with joint instability and is more frequently observed in conjunction with other CVJ abnormalities, such as Chiari malformation type I (31). Given the similarly flattened joint configuration observed in our AAI-affected dogs, underlying instability may also be present. However, our assessment was limited to bony structures, and we cannot draw conclusions regarding the condition of ligamentous support or potential ligamentous laxity. Moreover, our study was based on static imaging; therefore, joint instability could not be evaluated and would require dynamic imaging modalities for further assessment.

In small and toy breed dogs, as in humans, multiple CVJ malformations frequently occur concurrently, likely due to shared embryological origins. They refer to a group of structural abnormalities affecting the region where the skull base and upper cervical spine meet, potentially leading to neurological and biomechanical complications (21, 34, 35). In our study, AAI in dogs was accompanied by a more shallow conformation of the atlanooccipital joint, another region of the CVJ, supporting an association between AAI and other CVJ anomalies. The more shallow conformation of the atlantooccipital joints in the AAI group may be interpreted as a certain degree of joint dysplasia. Which is, in general, a developmental disorder affecting the bony structures that form a joint. In general, the incongruence in dysplastic joints leads to abnormal joint mechanics and progressive wear of the articular cartilage. As the bone structures become increasingly deformed, normal joint function is compromised, promoting further degeneration. Over time, these pathological changes culminate in the onset of clinical symptoms such as pain, reduced mobility, and joint instability (34). However, as our study evaluated the dogs at only a single time point, no conclusions can be drawn regarding potential progressive changes over time.

In the lateral atlantoaxial joint, the depth-to-length ratios of both the caudal articular surface of the atlas (Fovea articularis caudalis atlantis) and the cranial articular surface of the axis (Facies articularis ventralis axis) were generally lower than those observed in the atlantooccipital joint in both the sagittal and dorsal planes, indicating a generally flatter or more shallow conformation of the lateral atlantoaxial joints. This is consistent with its biomechanical role as a pivot joint, that primarily facilitates rotational movement, but also permits small amounts of flexion-extension and lateral bending, which rely on relatively flat, gliding lateral joint surfaces rather than deeply congruent joint surfaces (12, 24).

Although some significant differences were observed between the AAI and control groups in the lateral atlantoaxial joints, the results were partially contradictory regarding joint conformation. In the sagittal plane, the Fovea articularis caudalis atlantis appeared more curved in the AAI group compared to controls, while the Facies articularis ventralis axis did not differ significantly. Conversely, in the dorsal plane, the Fovea articularis caudalis atlantis was shallower and the Facies articularis ventralis axis more curved in AAI-affected dogs. These apparently inconsistent findings must be interpreted with caution due to the small absolute values of the measurements—particularly depth, which was frequently less than 1 mm—and the relatively large standard deviations measured on CT images with a spatial resolution in the *z*-axis of 0.4 mm, all of which introduce a degree of uncertainty. It may be assumed, that plane- and direction-dependent variations in joint surface curvature may be more complex in the lateral atlantoaxial joints reflecting combined rotational and translational movements compared to the biaxial ellipsoid atlantooccipial joint (34, 35).

Limitations of this study were its retrospective design, the relatively small sample size and the use of cadavers in the control group. The relatively small sample size in both the AAI and control groups limits the statistical power of the study, increasing the risk of type II errors—that is, failing to detect true differences between groups. With a limited number of subjects, the ability to generalize findings to the broader population of small breed dogs is reduced. Additionally, the small sample size may have contributed to the relatively large standard deviations observed in several measurements, thereby affecting the precision and reliability of the morphometric comparisons. Post-hoc sample size calculations indicated that the study was adequately powered to detect the moderate-to-large between-group differences observed in the atlantooccipital joint in the sagittal plane, but that larger samples would be required to reliably identify smaller effects, particularly for the Facies articularis ventralis axis. Future studies with larger, more diverse populations are needed to validate these findings and strengthen the evidence base. Additionally, all measurements were performed by a single observer, which may introduce interpretation bias. However, previous studies in human medicine have demonstrated good interobserver agreement and intraobserver reproducibility for similar CT-based morphometric measurements of the atlantooccipital joint (36). The inherently flat configuration of the atlantoaxial lateral joint surfaces resulted in very small depth-to-length ratios and made precise measurements technically challenging on reconstructed CT images with a resolution of 0.4 mm in the *z*-axis, increasing the risk of measurement inaccuracies. Moreover, measurements were obtained in a single central plane of the joint, representing only a limited cross-section and not the full articular surface. Future studies employing three-dimensional imaging and reconstruction techniques would allow for a more comprehensive evaluation of joint morphology in AAI-affected dogs. Another limitation was the use of cadavers in the control group and live dogs in the AAI group. Cadavers were scanned in a standardized head–neck position, which facilitated consistent joint orientation; however, postmortem soft-tissue relaxation and the absence of physiological loading may alter joint orientation and may thereby introduce bias when comparing with live dogs. Given that the parameters evaluated were based on bone morphology, it is unlikely that post-mortem changes significantly influenced the depth and length measurements. More critically, the present study focused solely on osseous structures, excluding contributions from soft tissues such as synovial structures or ligaments. These components likely play a major role in joint function and stability and may explain why the observed bony morphology does not fully reflect the complexity of joint biomechanics.

In conclusion, significant differences have been found in the atlantooccipital joints, where the joint surfaces were flatter in the AAI group compared to the control group. Therefore, atlantoaxial instability should not be considered in isolation but rather as part of the broader complex of CVJ malformation.

In the future, improved CT resolution or three-dimensional representation could enhance the reliability of measurements. Future studies should ideally involve a larger sample size and possibly alternative measurement methods to confirm and expand upon these findings. Additional research is needed to fully elucidate the pathophysiological effects of morphometry on atlantoaxial instability in these patients.

## Data Availability

The raw data supporting the conclusions of this article will be made available by the authors, without undue reservation.
