# Supplementary material for: Morphometric study of the atlantooccipital and the lateral atlantoaxial joints in small breed dogs with and without atlantoaxial instability
Source: Front Vet Sci. 2026 Feb 9;12:1699281. doi: 10.3389/fvets.2025.1699281 (PMC12926163; doi:10.3389/fvets.2025.1699281)
Supplement: Supplementary file 1 [file Table_1.docx]

Supplementary Material

# Supplementary Tables

**Supplementary Table 1** Median values for the right and left sides, with p-values from Wilcoxon signed-rank tests for related samples comparing depth-to-length ratios of the left and right atlantooccipital and lateral atlantoaxial joints, separately for control (Control) atlantoaxial instability (AAI) groups. (CO: Condylus occipitalis, FACraAt: Fovea articularis cranialis atlantis, FACauAt: Fovea articularis caudalis atlantis, FAVenAx: Facies articularis ventralis axis, D/L: depth-to-length, Sag: sagittal plane, Dor: dorsal plane)

|  | **Control** | | | **AAI** | | |
| --- | --- | --- | --- | --- | --- | --- |
|  | **Median Right** | **Median Left** | **p-value** | **Median Right** | **Median Left** | **p-value** |
| **CO D/L Ratio Sag** | 0.40 | 0.44 | 0.31 | 0.30 | 0.32 | 0.33 |
| **CO D/L Ratio Dor** | 0.20 | 0.21 | 0.83 | 0.16 | 0.14 | 0.026 |
| **FACraAt D/L Ratio Sag** | 0.34 | 0.35 | 0.52 | 0.21 | 0.19 | 0.66 |
| **FACraAt D/L Ratio Dor** | 0.08 | 0.07 | 0.81 | 0.06 | 0.07 | 0.53 |
| **FACauAt D/L Ratio Sag** | 0.05 | 0.06 | 0.36 | 0.13 | 0.08 | 0.66 |
| **FACauAt D/L Ratio Dor** | 0.10 | 0.10 | 0.19 | 0.07 | 0.07 | 0.86 |
| **FAVenAx D/L Ratio Sag** | 0.18 | 0.18 | 0.31 | 0.19 | 0.19 | 0.66 |
| **FAVenAx D/L Ratio Dor** | 0.13 | 0.12 | 0.13 | 0.18 | 0.14 | 0.41 |

**Supplementary Table 2** Post-hoc sample size calculations for the control (Control) and atlantoaxial instability (AAI) groups for depth-to-length ratios of the atlantooccipital and lateral atlantoaxial joints. (CO: Condylus occipitalis, FACraAt: Fovea articularis cranialis atlantis, FACauAt: Fovea articularis caudalis atlantis, FAVenAx: Facies articularis ventralis axis, D/L: depth-to-length, Sag: sagittal plane, Dor: dorsal plane)

|  | **Control** | **AAI** |
| --- | --- | --- |
| **CO D/L Ratio Sag** | 7 | 14 |
| **CO D/L Ratio Dor** | 13 | 25 |
| **FACraAt D/L Ratio Sag** | 4 | 7 |
| **FACraAt D/L Ratio Dor** | 68 | 135 |
| **FACauAt D/L Ratio Sag** | 11 | 22 |
| **FACauAt D/L Ratio Dor** | 13 | 25 |
| **FAVenAx D/L Ratio Sag** | 673 | 1346 |
| **FAVenAx D/L Ratio Dor** | 26 | 51 |
